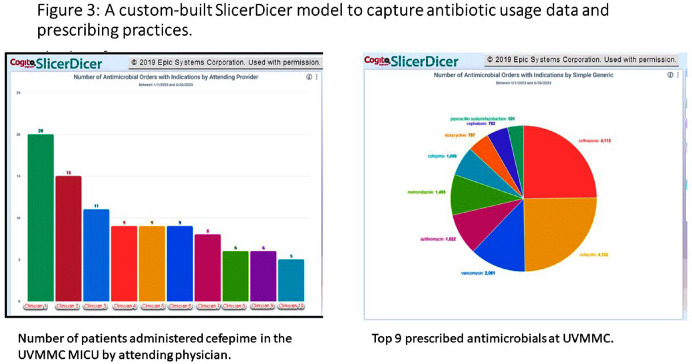# An “Epic” Journey to Improve Antimicrobial Stewardship

**DOI:** 10.1017/ash.2024.340

**Published:** 2024-09-16

**Authors:** Lindsay Smith, John Ahern, Lisa Lapin, Thyleen Tenney

**Affiliations:** University of Vermont; University of Vermont Medical Center; The University of Vermont Health Network

## Abstract

**Background:** Antimicrobial stewardship programs rely heavily on the electronic medical record (EMR) to carry out daily activities, make interventions, optimize patient care, and collect data. In 2019 the University of Vermont Medical Center transitioned from using a third party platform to the Epic (Verona, WI, www.epic.com) Bugsy module for antimicrobial stewardship. **Method:** We have spent the past 4 years optimizing the Epic foundation to match our institutional antimicrobial prescribing guidelines, susceptibility patterns, and build reports to extract actionable data. **Result:** During the build process, we readily identified three areas needed for customization: (1) Empiric, definitive, and prophylactic indications of use for all antimicrobials based on our hospital’s internally published books “Guide to Antimicrobial Therapy for Adults” and “Guide to Antimicrobial Therapy for Pediatrics” (figure 1); (2) An on-demand report to capture all patients with new administrations of antimicrobials in the preceding 72 hours, that includes ordering clinician, stop date of therapy, and indication (figure 2); and (3) A unique, custom-built slicer-dicer report to capture high-level data on how each antimicrobial is being prescribed by indication, dose, route of administration, ordering clinician, attending physician, and department (figure 3). **Conclusion:** We have built a system where we can readily identify patients that are receiving antimicrobials both within and outside of institutional guidelines and know the ordering clinician to contact to provide in-the-moment feedback. We can also collect retrospective data to know which antimicrobial agents were prescribed for all infectious syndromes. These three institutional customizations have provided invaluable information to improve patient care.

**Figure:**
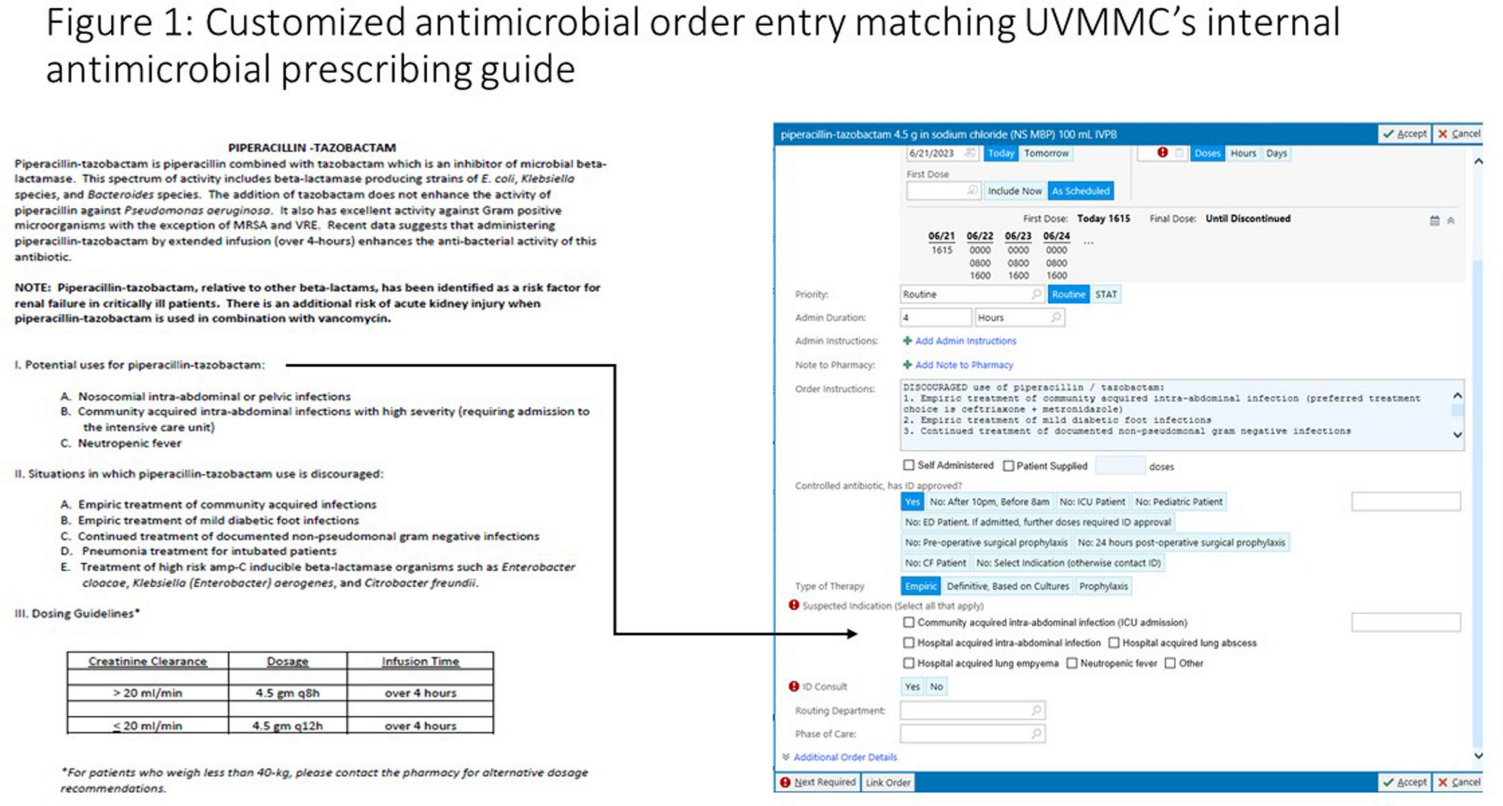

**Figure:**
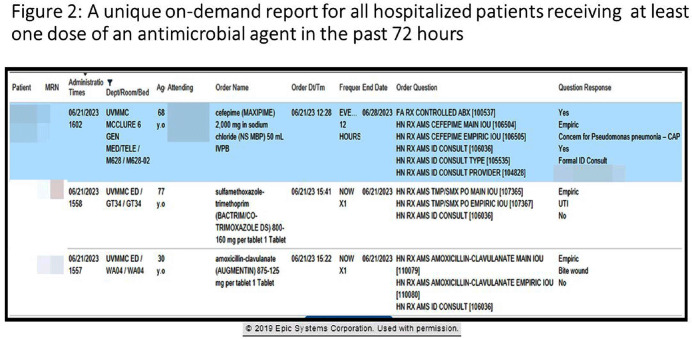

**Figure:**